# The complete chloroplast genome sequence of the *Canna edulis* Ker Gawl. (Cannaceae)

**DOI:** 10.1080/23802359.2020.1775512

**Published:** 2020-06-11

**Authors:** Qianglong Zhu, Lijuan Cai, Huiying Li, Yu Zhang, Wenzhen Su, Qinghong Zhou

**Affiliations:** a Department of Horticulture, College of Agronomy, Jiangxi Agricultural University, Nanchang, P.R. China; b Nanchang Business college, Jiangxi Agricultural University, Nanchang, P.R. China

**Keywords:** *Canna edulis* Ker Gawl, chloroplast genome, edible canna

## Abstract

*Canna edulis* Ker Gawl. is an essential traditional tuber crop used for fresh consumption and to isolate starch in some tropical and semitropical regions. The complete chloroplast genome sequence of *C. edulis* has been determined in this study. The total genome size is 164,650 bp in length and contains a pair of inverted repeats (IRs) of 27,278 bp, which were separated by large single-copy (LSC) and small single-copy (SSC) of 91,421 bp and 18,673 bp, respectively. A total of 131 genes were predicted including 86 protein-coding genes, 8 rRNA genes and 37 tRNA genes. Further, maximum-likelihood phylogenetic analysis revealed that *C. edulis* belongs to Cannaceae in Zingiberales. The chloroplast genome of *C. edulis* is first complete genome sequence in Cannaceae and would play a significant role in the development of molecular markers in plant phylogenetic and population genetic studies.

*Canna edulis* Ker Gawl. commonly known as ‘edible canna’, is an essential traditional tuber crop used for fresh consumption and to isolate starch in some tropical and semitropical regions (Pérez and Lares 2005). The edible canna that contain abundant starch and characterized by large granules, high amylose content, and high viscosity, is commonly used in animal fodder, cakes, noodle, and purple dye (Zhang et al. 2013). *Canna edulis* can also be used as garden ornamentals for the treatment of industrial waste waters through constructed wetlands (Sandoval et al. 2019). The species was origin from Central and South America and distributed in Europe, North America, and many tropical regions of the world (Gupta et al. 2013). *Canna* includes 8–10 wild species and over 1000 hybrids (Biswabijayinee et al. 2008), it is difficult to characterize and identify diverse *Canna* germplasm by traditional morphological characters, which hinders the excavation and utilization of *Canna*. Chloroplast genome is a molecular resource for developing DNA markers for plant identification (Ganie et al. 2015). However, to the best of our knowledge, there are no reports that the chloroplast genome of *C. edulis* was taken as a molecular resource. Thus, the goal of this study is to sequence the chloroplast complete genome of *C. edulis* with the hope of promoting the studies on the species identification, germplasm exploration, and phylogenetic relationships.

Sample of *C. edulis* (JT31) was planted in Jiangxi Agricultural University (28°45′53″N, 115°49′41″), Nanchang, China. The genomic DNA was extracted from the fresh leaves of *C. edulis* using the CTAB method as previously described (Porebski et al. 1997). About 15 μg extracted DNA was used for library construction and genome sequencing on the HiSeq X Ten Platform (BGI, Shenzhen, China). After sequencing and base quality control, a total of 1 Gb of sequence data in fastq format was obtained. The draft genome sequence was assembled by using the Plasmidspades.py in SPAdes 3.14.1 (Bankevich et al. 2012), BlastN v2.7.1, and Gapcloser v1.12-r6. Contigs representing the chloroplast genome were then retrieved, ordered, and incorporated into a single draft sequence by comparison with the chloroplast genome of *Zingiber officinale* (NC_044775.1) using BlastN. The gaps in the chloroplast single draft sequence were closed by using GapCloser and the complete genome was confirmed and manually corrected by PE read mapping. Finally, the complete genome sequence was annotated using CPGAVAS2 (Shi et al. 2019), and manually checked and corrected by Sequin.

The complete chloroplast genome of *C. edulis* (MN832865) is 164,650 bp in length with 36.22% GC contents, and exhibits a typical quadripartite structure, consisting of a pair of IRs (27,278 bp) separated by the LSC (91,421 bp) and SSC (18,673 bp) regions. There is a total of 131 genes, including 86 protein-coding genes, 8 rRNA genes and 37 tRNA genes; six of the protein-coding genes, six of the tRNA genes and four rRNA genes are duplicated within the IRs.

To determine the phylogenetic position of *C. edulis*, a phylogenetic tree including other 30 species in Zingiberales was constructed by the maximum-likelihood (ML) method using the program MAFFT v7.407 (Nakamura et al. 2018) and MEGA v10.0.4 (Kumar et al. 2018). The tree showed that *C. edulis* belonged to Cannaceae (Figure 1), and is closer to *Ravenala madagascariensis* and *Heliconia collinsiana* in Zingiberales. The conclusions further support the previous research results (Shetty et al. 2016).

**Figure 1.**
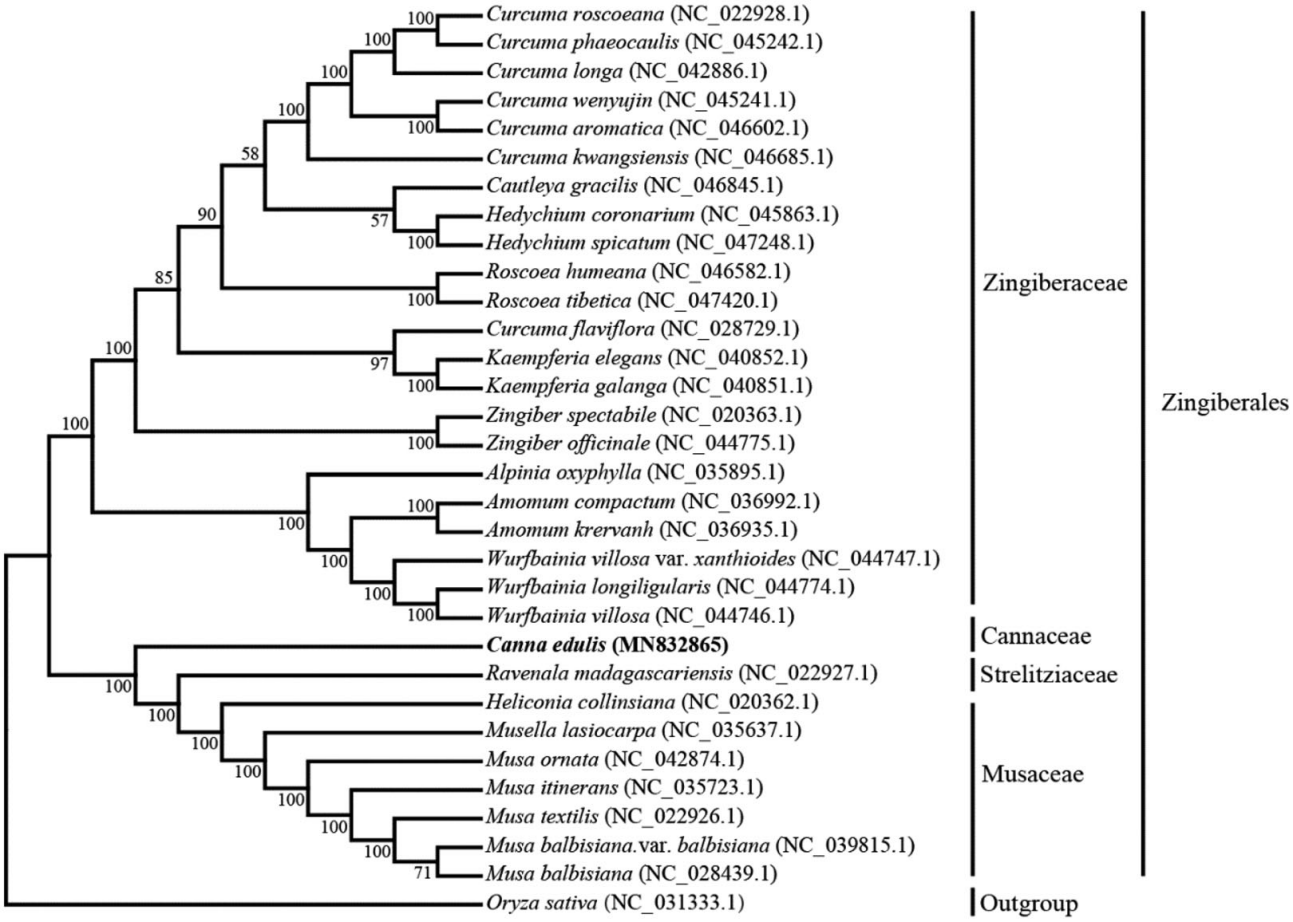
Phylogenetic tree showing relationship between *C. edulis* and other 30 species in Zingiberales, *Oryza sativa* (NC_031333.1) was taken as the outgroup. Phylogenetic tree was constructed based on the complete chloroplast genomes using the maximum-likelihood (ML) with 1000 bootstrap replicates. Numbers in each the node indicated the bootstrap support values.

## Data Availability

The data that support the findings of this study are openly available in GenBank at https://www.ncbi.nlm.nih.gov/nuccore/MN832865.1/, reference number MN832865.
